# Revisiting the sub-mandibular approach with a modified incision

**DOI:** 10.11604/pamj.2020.36.171.24289

**Published:** 2020-07-10

**Authors:** Namdeo Prabhu, Zafar Ali Khan, Rakhi Issrani

**Affiliations:** 1 Department of Oral & Maxillofacial Surgery and Diagnostic Sciences, College of Dentistry, Jouf University, Sakaka, Kingdom of Saudi Arabia,; 2 Department of Preventive Dentistry, College of Dentistry, Jouf University, Sakaka, Kingdom of Saudi Arabia

**Keywords:** Incision, mandibular angle, sub-mandibular

## Abstract

Angle of mandible has been long plagued throughout the history for being one of the most common places of facial bone fracture and also related complications if not treated properly. Hereby, two cases of fracture of angle of mandible those were surgically treated with an aim to modify existing extra-oral approaches specifically for treating the mandibular angle fractures for the purpose of open reduction and internal fixation so as to have a good access combined with an aesthetic and inconspicuous scar postoperatively are described. Also, an attempt is made to add one more type of incision to many existing ones so that future surgeons can choose over which best suits their procedure and as an addition to the ever-increasing literature. There is no doubt that meticulous surgical procedure and experience is needed to identify the structures, to stay in the plane of desire and work in comparatively bloodless field. Hence this approach can be bit challenging for newer surgeons. Nonetheless, the result after the closure of the field is quite rewarding.

## Introduction

Of all the facial landmarks which give defining character to the facial structure, there is no doubt that gonial angles or more commonly known as angle of mandible stands out in terms of lateral extension of lower third of face and supported by strong sturdy bone. Angle of mandible has been long plagued throughout the history for being one of the most common places of facial bone fracture and also related complications if not treated properly. To avoid all these complications, open reduction and internal fixation supplemented by an extra-oral approach has been a norm in recent times [1]. Being so aesthetically important landmark and carrying the burden of one of the most commonly fractured bone and related complications perhaps the incision line and surgical approach for open reduction of mandibular angle is the cornerstone of the treatment planning.

Number of approaches have been described, some similar to the design [2] that is used in the current case and others including postramal (Hinds), submandibular (Risdon), transbuccal trocar and intra-oral approaches with numerous modifications [3-5]. But each one carries its own complications and difficulties and hence, in authors´ view, an incision design with more specialised approach is needed or at least present one more option to aesthetically designed incisions. Against this backdrop, the following cases were surgically treated with an aim to modify existing extra-oral approaches specifically for treating the mandibular angle fractures for the purpose of open reduction and internal fixation so as to have a good access combined with an aesthetic and inconspicuous scar postoperatively. Also, an attempt is made to add one more type of incision to many existing ones so that future surgeons can choose over which best suits their procedure and as an addition to the ever-increasing literature.

## Patient and observation

**Figure 1:**
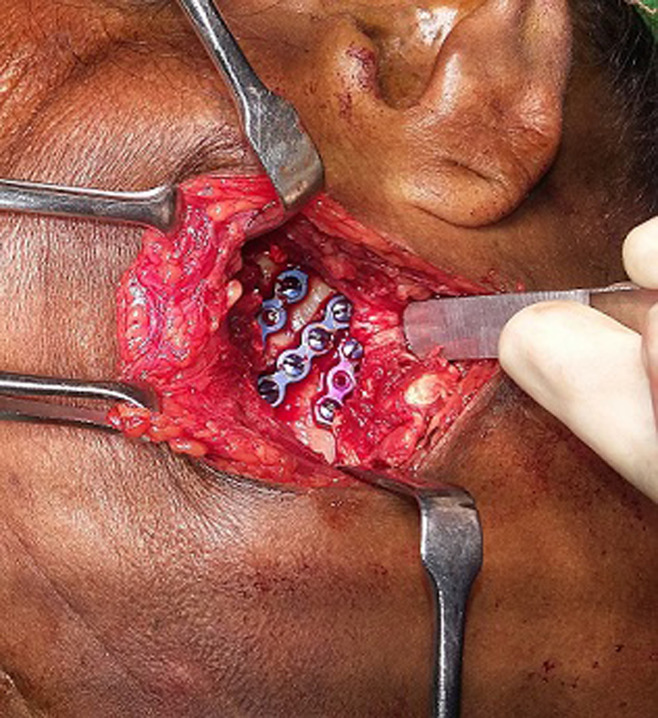
intra-operative pictures show reasonable access to the fracture fragment in a 28-year old female patient

**Case presentation 1:** a 28-year-old female patient was diagnosed with comminuted mandible fracture on left side. She sustained the injury after falling from a height when she climbed up a tree for harvesting fruits. She presented with fracture in symphysis region also. She was opted for open reduction as the wound was infected when she reported to the hospital and closed reduction would not have been suitable for the type of fracture. Patient was treated under general anaesthesia and surgical procedure was carried out as described. Intra-operative pictures show excellent access to the fracture fragment which was enough to reduce them and place 3 miniplates (Figure 1).

**Figure 2:**
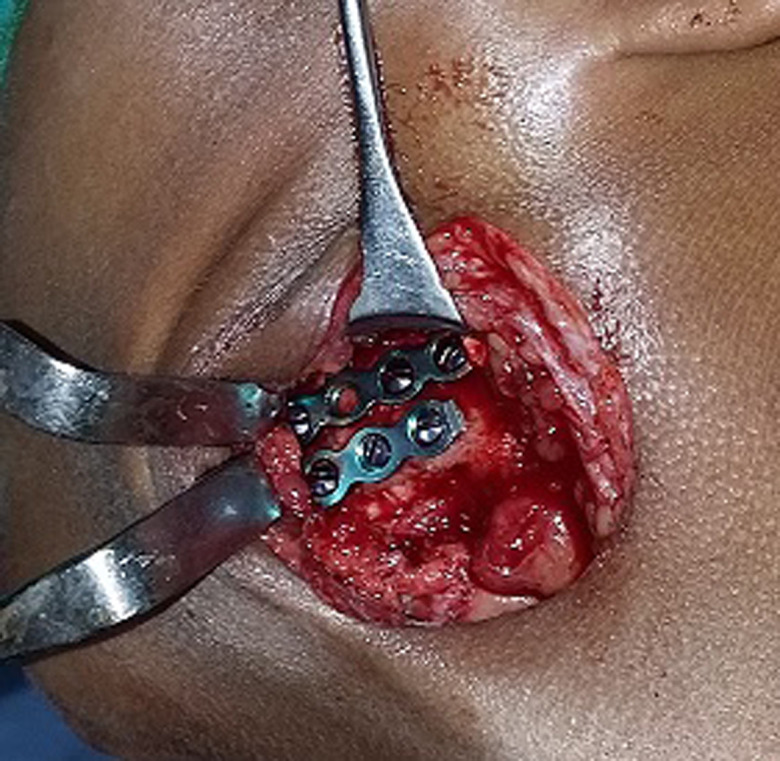
satisfactory access for placement of 2 miniplates in a 21-year-old male

**Case presentation 2:** a 21-year-old male suffered isolated mandibular angle fracture following a road traffic accident. Patient in this case wanted early return to the work and also on CT scan a torsional deformation was seen on the fracture fragments. Hence the decision to do open reduction was made. Incision designing and access ad procedure was carried out as described. Access was satisfactory and 2 miniplates were placed and fixation achieved (Figure 2).

**Surgical procedure:** both the above-mentioned patients were treated under general anaesthesia. Thiopentone sodium was used for induction and patients were intubated nasally. Oral cavity was kept free to achieve maxillo-mandibular fixation and achieve the occlusion. Isoflurane and/or sevoflurane were used for maintenance. Surgical site and incision line were marked with a marking ink before commencing the procedure. Thereafter the marked line was infiltrated using lidocaine with adrenaline in 1: 1000 concentrations. Incision remains the key of this approach as aesthetics has been given prime importance to the designing of this surgical access. Care has to be taken that incision follows the curvature of the angle of the mandible and suture line has to be visualized beforehand such that it is hidden completely in the shadow of the angle. Incision can be said to be combination of postramal Hind´s incision and submandibular Risdon´s incision but to the fact that it remains closer to the angle of the mandible, around 1 cm away from the bony landmark completely in the shadow of the mandible. Sharp incision with a 15 number blade is imperative to avoid any jagged incision line. In the first stroke only skin and subcutaneous tissue should be visible and skin flaps hold tightly away with skin hooks, which gives clear view of the subcutaneous tissue. Platysma muscle once seen can be dissected easily with a pair of scissors and blade with care that the superficial layer of deep cervical fascia is left undisturbed.

**Figure 3:**
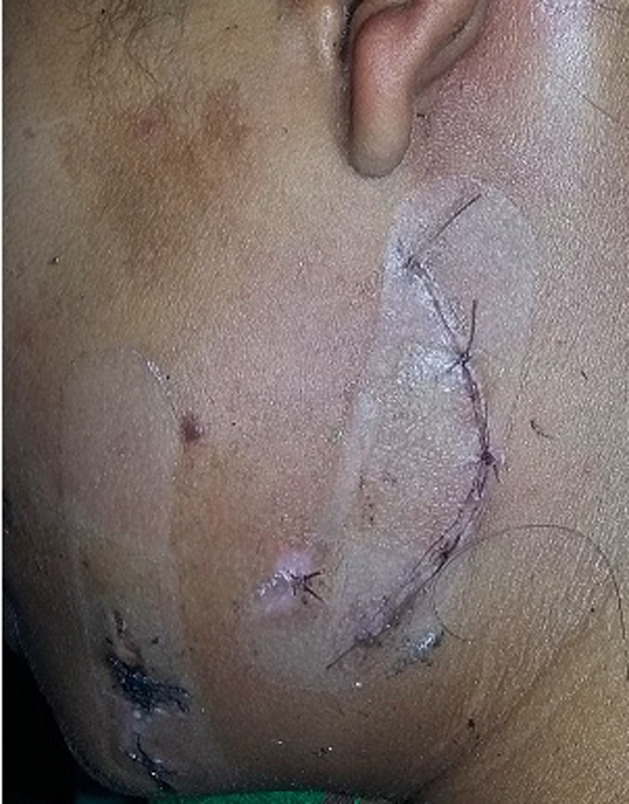
excellent wound healing with a fine-looking scar within the shadow of the mandibular angle on the 7^th^post-operative day in treated case 1

**Figure 4:**
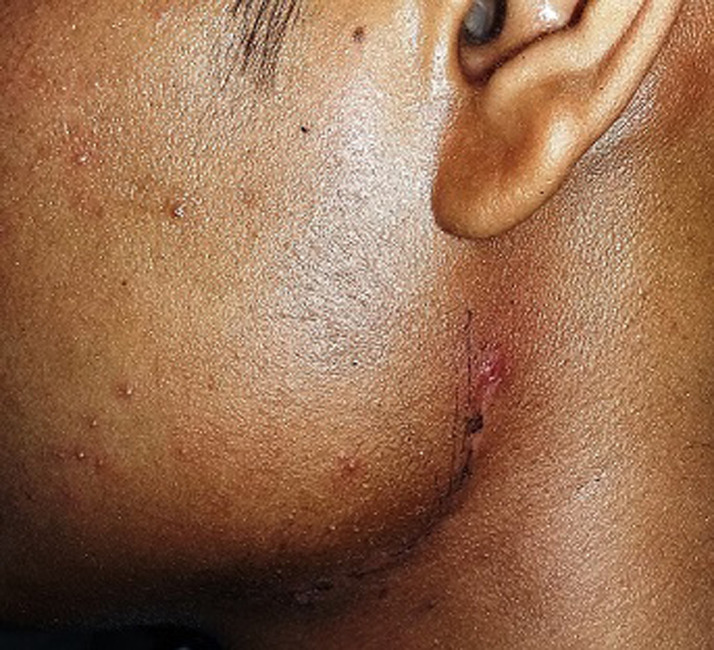
excellent wound healing with a fine-looking scar within the shadow of the mandibular angle on the 7^th^post-operative day in treated case 2

Further careful progression and perhaps the most challenging step is dissecting through the deep cervical fascia which is important because the facial nerve, facial artery and facial vein will be lying deep to the fascia and hence absolute care has to be taken while incising the deep fascia. Immediately after incising the deep fascia, it is of utmost importance to identify the facial nerve with the nerve stimulator and retracting it in the upper flap. Subsequently, facial artery and vein should be identified, and if necessary, should be ligated and retracted to expose the underlying pterygo-masseteric sling. Once the pterygo-masseteric sling is visible then it becomes fairly easy procedure, whereby sharp incision on the sling can be given to expose the mandibular angle and this plane is fairly avascular with not much bleeding. Once the angle is exposed, fracture fragments can be easily manipulated to achieve reduction. Miniplates can be placed thereafter and fixation achieved. After irrigating the surgical site, closure is achieved in layers to define anatomic spaces. Great care was taken to achieve skin closure wherein subcutaneous horizontal mattress suturing was done to achieve the skin approximation which gives excellent post-operative scar. In both the treated cases, no suture tracks were noticed and a linear scar within the mandibular angle shadow was seen which was almost inconspicuous at the conversational distance (Figure 3, Figure 4).

## Discussion

Submandibular Risdon´s incision has been the workhorse in the approach to the mandibular body, ramus and submandibular region and there are not many or virtually no approaches to overcome the difficulties of this approach [6]. Even though the importance of Risdon´s incision cannot be denied in the submandibular region, its use in the specialised cases of mandibular angle fracture is somewhat guarded in authors´ view, due to the fact that longer incision leads to decreased aesthetic outcomes. The basic principle of the Risdon´s incision is to avoid the mandibular branch of the facial nerve (MBFN) completely by placing the incision in the cervical crease at least 2cm away from the mandibular border [6]. Thereby, the MBFN which is left in the upper flap can be directly retracted and there are fewer chances of damage. But these manoeuvres have some serious aesthetic and surgical sequela. Aesthetically the incision line is absolutely conspicuous because of 2 reasons, firstly that it is placed in the neck and is visible at the conversing distance, secondly the length of the incision has to be large as tissue retraction needs to be done to greater extent as the incision is placed far away from the area of interest. Surgically, if we consider, MBFN is not identified and hence the procedure blindly relies on the assumption that MBFN will be in the upper flap. Other concern is the long way of dissection to reach the area of interest which will ultimately lead to longer incision line and greater retraction of the flap leading to greater nerve traction.

In the current surgical approach, it was noticed that by placing an incision high up, all these complications can be avoided to great extent when the primary concern is only to approach the mandibular angle. Identifying MBFN and retracting it in upper flap makes this procedure no more a blind procedure. Also, the length of the incision is greatly reduced as it is placed nearer to the area of interest and completely hidden in the shadow of the mandible making it almost inconspicuous at the conversing distance. Postramal (Hinds) incision and pre-auricular approaches has also been used over the years to approach the mandibular angle region albeit with some modifications [7]. These approaches pose the challenge that inferior border of the mandible is difficult to explore and secondly many times we need a trans-parotid route. Nonetheless, the lower placement of postramal incision if combined with horizontal incision akin to higher placed Risdon´s incision as the authors´ describes would take care of these concerns with an added benefit that the whole incision line lies in the shadow of the mandible that gives a fine-looking post-operative result.

Other modalities like transbuccal trochar system is again an indispensable tool, but greatly challenged by the fact that it severely limits the visualization of the fracture site [8]. It makes the manipulation of bony fragments difficult and fixation screws and plates are highly difficult to place in the area of interest. Also, it has to be supplemented by the intra-oral incision to deliver the hardware and visualize the fracture site. Moreover, the fact remains that MBFN is not recognized and stab incisions to place the cannula is expected to miss any important structures. In the current surgical approach, an attempt has been made to avoid all these challenges. Nevertheless, there are two challenges in the current approach. Firstly, it needs high level of experience to identify all the structures and isolating them needs greater dexterity so that expected outcomes are not left to chances. Secondly, there is a need to extend the incision in case of comminuted fractures which are extending in the body region which will lead to the scar that is quite visible. Yet in the current approach it was observed that it gives optimum results when we considered the challenges in other approaches. Also, we expect to come up with new modifications in the future to realize an almost ideal incision when the primary concern is treating the fractures in the mandibular angle region.

## Conclusion

There are many incisions which have stood the test of time and have been widely accepted. But oral and maxillofacial surgery has been one of newer surgical specialties and its needs and demands have been different. Giving newer techniques specially tailored for the surgical procedures that are recently performed is the need of the time. This field stands at the threshold of the aesthetic surgery and hence it is imperative to strive towards newer techniques that give better post-operative results in a similar way as in the cases described here.
